# Manual of the Diseases of the Eye

**Published:** 1911-10

**Authors:** 


**Manual of the Diseases of the Eye.** By **Charles H. May,** M.D., Chief of Clinic and Instructor in Ophthalmology, College of Physicians and Surgeons, Medical Department, Columbia University, 12mo., 407 pages, with 362 original illustrations. New York: William Wood & Co., 1911. (Cloth, $2.00.)

May's very excellent Manual of the Diseases of the Eye shows every evidence of having been thoroughly revised in its seventh edition. The use of salvarsan, the Lagrange operation, the Eldridge-Green theory of color vision, and tubercular medication are among the recent medical advances to which reference has been made. An important addition is a chapter on Ocular Manifestations of General Diseases.

The colored plates are especially good. The other illustrations are better than usually encountered in similar books, but there are a few whose omission would enhance the value of the work.

The book is heartily recommended to students. Its title should replace in our college catalogues those of the more comprehensive volumes usually advised in the course of study. Also there is no better eye book for the general practitioner, and particularly for him who is so situated that he has not always available the friendly counsel of a specializing brother.

A book that has gone through seven editions in eleven years and has been translated into six foreign languages must indeed supply a want. H. W. C.

